# Effect of a hospital-based oral health-education program on Iranian staff: evaluating a theory-driven intervention

**DOI:** 10.1186/s12909-020-02435-4

**Published:** 2021-01-06

**Authors:** Bahram Armoon, Mohsen Yazdanian, Peter Higgs, Hormoz Sanaei Nasab

**Affiliations:** 1 Social Determinants of Health Research Center, Saveh University of Medical Sciences, Saveh, Iran; 2 Research Center for Prevention of Oral and Dental Diseases, Baqiyatallah University of Medical Sciences, Tehran, IR Iran; 3 Department of Public Health, School of Psychology and Public Health, La Trobe University, Melbourne, Australia; 4 Health Research Center, Life Style Institute, Baqiyatallah University of Medical Sciences, Tehran, Iran; 5 Health Education Department, Faculty of Health, Baqiyatallah University of Medical Sciences, Tehran, Iran

**Keywords:** Oral health education, Theory of planned behavior, Staff, Tehran

## Abstract

**Background:**

Tooth decay and periodontitis are among the most prevalent dental diseases globally with adverse effects on an individual’s general health. Recently the prevalence of dental caries has decreased significantly, but caries epidemiology remains a major problem in dental public health. This study investigated the impact of an oral health education intervention on Theory of Planned Behavior (TPB) variables, and whether changes in these variables persisted and were associated with changes in identified oral health behaviors at 2-month follow-up.

**Methods:**

This descriptive pre/post test study was conducted with 160 staff in the Baqiyatallah Hospital in Tehran. Six hospital wards were selected using a randomized multi-stratified sampling frame. The size for each cluster was calculated as 22 with each ward being allocated to either the intervention or the control arm of the study. Self-report questionnaires were used to evaluate socio-demographic factors, dental attendance as well as the constructs of the augmented TPB model (attitudes, subjective norms, perceived behavioral control intentions). The intervention was an educational program based on TPB constructs delivered via direct training to half the participants. The control group was provided with usual training only. The independent-samples T-test, Repeated-Measures one-way ANOVA, and matched T-test with the significance level set at *p* < 0.05 were applied.

**Results:**

Findings revealed significant variations between the two groups immediately after the educational intervention concerning the attitudes, subjective norms, perceived behavioral control, intentions to seek treatment, oral health behavior as well as decayed, missing, and filled teeth and bleeding on probing (*p*<0.001). Two months after the intervention, except for the brushing construct (*p* = 0.18), the differences between the two groups were all statistically significant (*p*<0.001).

**Conclusion:**

Our findings affirm the positive effect an oral health education program has on enhancing the attitudes, subjective norms, perceived behavioral control, intentions and behavior of staff in this hospital. The results of our study confirm that developing and applying an educational intervention in accordance with the theory of planned behavior can lead to significant changes in the knowledge, attitudes, and behavior of hospital staff regarding preventing tooth decay.

## Background

Tooth decay and periodontitis are among the most prevalent dental diseases globally with documented adverse effects on an individual’s general health [1, 2]. Despite decreasing prevalence, dental caries epidemiology remains an important public health problem for dental and oral health research [3, 4]. Oral health problems are strongly associated with individual lifestyle factors such as diet and oral hygiene [5, 6] and the most influential factor reducing these and other individual health problems is preventive health behavior change [7].

The mainstream media has been found to both positively and negatively impact the health-related behaviors of the population [8]. Evidence suggests that effectiveness is increased when multiple interventions are used and when the target behavior is narrow [6]. Ensuring accessibility to health services and products is also essential if people are to be motivated to change behavior based on mainstream media messaging alone [9]. The development of policies that support opportunities for behavior change may motivate individual behavior change whereas not having enabling public health policies in place may support the development (or continuation) of unhealthy behaviors [10]. Public health messaging using the news and entertainment media also demonstrate a hopeful complementary strategy to enhancing awareness and communicating knowledge [11].

Considering education plays a major role in enhancing health behavior [12], it is evident that healthcare staff with adequate knowledge and skills in prevention of oral disease can improve oral health, especially of more marginalized groups [13]. Accordingly, the effectiveness of oral health education, can be assessed with the appropriate use of the theory and models.

The use of interactive audio-visual workshops has been shown to be more effective than traditional approaches in educating health professionals [14, 15]. Further understanding the utility of health education may also enhance the continuing health education service systems [16]. The theory of planned behavior (TPB) has been used to predict behavior change and intentions to change across a variety of disciplines, including advertising, public relations and in public health [17]. Previous studies have also applied this theoretical framework specifically to understand barriers for behavior change adherence in oral health education programs [18–20]. The TPB can improve interventions by determining particular information about participants [21]. The TPB is a useful framework for designing behavioral change interventions over which individuals are thought to have control [22]. Our study was designed with the assumption that a theory-driven intervention to enhance staff’ understanding of oral health prevention would increase the frequency of visits to the dentist and have other individual oral health benefits. Theory-driven interventions assume that changing behaviors is possible and that those changes, in turn, enhance positive future behaviors [19]. TPB is a well utilized social cognitive model showing how health-related behaviors can be adapted by individual intention [17]. In the oral health setting, it has been found that attitudes, social norms, and perceived behavioral control all contribute to the development of positive oral health behaviors [23]. Previous research has used TPB to predict staff intentions to control their sugary snack in-take behavior [19]. It also has been revealed that educational interventions can be beneficial for increasing oral health knowledge and attitudes toward using less sugar and more frequent brushing of teeth [24]. The TPB constructs can also have a positive effect on intended behavior at the end of a sugar snack intervention program. Clearly increasing knowledge can also influence the intention to enact behavior change [25, 26].

An important approach to improving overall oral health is to increase knowledge and understanding of available interventions [27]. At its core health education is designed to eliminate certain negative behaviors and promote alternate positive ones [28]. Further, the positive effects of health education on improving oral health behavior have been well documented [29–31]. The first phase of designing a health-related behavior change intervention is choosing an appropriate model. TPB is a well utilized behavioral change model and it is useful because it contains most aspects of the psychological determinants and predictors of oral and dental health associated behavior change [17]. TPB indicates that behaviors which are associated with health are predictable by what is termed the ‘intention construct’ [17, 32]. TPB also includes attitudes and orientations, subjective norms, and perceived behavioral controls. Attitudes to behavior change are defined by individual beliefs about the results of the health-related behavior as assessed by the potential outcome. A subjective norm is the opinion of people that are important in their life and may influence them to behave in a certain manner, assessed by the level of adaptation with that effect. Perceived behavioral control is the opinion of people that certain factors simplify or prevent action, assessed by the perceived control of them over these factors [33].

To date, there are a limited number of studies applying the TPB to oral health-related behavior in adults [34, 35] with many of using a cross-sectional design. Of the few prospective studies completed the follow-up periods have been less than 2 months [32, 36]. The benefits of maintaining healthy behaviors over time highlight the advantage of using TPB prospectively [37, 38]. As few studies have investigated the TPB determinants associated with oral health improvements prospectively [39–41] the present study was designed to assess an oral health-related behavior intervention for hospital staff using the TPB framework.

## Methods

### Study design and sampling

In our descriptive pre/posttest intervention, the study population included all staff in the Baqiyatallah Hospital in Tehran. The sample size was calculated based on previous research [42] which considered Z1-α/2 = 1.96, power = 95%, and with an attrition rate of 10%. The sample included 160 staff (80 participants in the intervention arm and 80 in the control group). A multi-stratified randomized sampling method was applied. Six wards of the hospital were chosen. The best size for each cluster was calculated as twenty-two. Hospital wards were allocated to either the experimental or control groups.

### Cluster determination

The number of clusters was calculated based on an existing formula [43], the alpha was obtained 5%, the difference 20, inter-cluster and the intra-cluster distances were 5 respectively, and the *n* = 22 and 6 clusters were obtained.

The stratified random sampling was used and participants were included in the study were anonymous, and each sample was randomly selected for each cluster.

The inclusion criteria were: interested in taking part in the study, more than 2 years’ work experience in the same hospital and not currently working as an oral health professional. Individuals who were not available or absent from the educational program, lacked interest to complete or provided incomplete responses to the questionnaires were excluded from the research. The data were collected between March and June 2019.

### Instrument

Data were collected using a previously published TPB-based questionnaire [42]. The instruments were assessed and checked again for validity and reliability for use in this setting. The validity of the questionnaire was assessed by an expert panel (*n* = 14) and the reliability was assessed using a test-retest internal consistency with a sub sample of thirty staff.

### Content validity

The experts were asked to review the questionnaire and evaluate each item considering 4 criteria including relevancy, clarity, simplicity, and necessity [44]. The content validity ratio (CVR) was assessed according to replies to the necessity of questions (nE) and the following formula of CVR was applied: CVR = (nE-N/2)/ (N/2). To specify the cut-off point for CVR, the Lawshe’s table was used [45]. Considering the Lawshe for 10 professionals, the minimum required CVR for each item was 0.74. The content validity index (CVI) was assessed based on the approach of Waltz and Bausell [46]. CVI for each item was calculated by dividing the number of professionals who classified the items as compatible for each criterion (relevancy, clarity, and simplicity) to the total number of professionals responding. The average value of three criteria as the total CVI for each item was applied. The minimum required value of CVI for each item was 0.79.

To determine the reliability of the internal consistency tool, Cronbach’s alpha method was used with 40 samples. The results demonstrated that Cronbach’s alpha coefficient for tool structures varied from 0.71 to 0.93.

To determine the stability of the tool, the method was the test, re-test and with a 41- samples with the time interval of 2 weeks were used.

Intraclass correlation coefficient (ICC) was used to analyze the results and showed that ICC for tool structures varied from between 0.69 to 0.79 —the total tool was 0.72. (Table 1).

**Table 1:** Internal Consistency and ICC^a^ Coefficients for TPB^b^-based Questionnaire (*n* = 40)

Construct	Cronbach’s alpha value (95% CI)	ICC (95% CI)
*Attitude*	0.88 (0.64–0.93)	0.69 (0.7–0.68)
*Subjective Norms*	0.93 (0.81–0.98)	0.71 (0.68–0.74)
*Perceived Behavioural Control*	0.71 (0.49–0.83)	0.74 (0.66–0.77)
*Intention*	0.89 (0.78–0.94)	0.79 (0.69–0.87)
*Total*	0.91 (0.81–0.95)	0.72 (0.68–0.75)

^a^Intraclass correlation coefficient

^b^Theory of Planned Behavior

The TPB-based questionnaire contained three sections. The first consisted of 10 items including demographic information such as age, sex, medical insurance, education, income status, and marital status. The second and third sections included 17 TPB items, documenting oral health behavior such as brushing, flossing, and visits to the dentist. The fourth section documented DMFT and applied the bleeding on probing (BOP) index (see Table 2).

**Table 2:** Description of Study Instrument (Source: *Modified from Ebrahimpour* et al *questionnaire*)

***Construct***	***No. of Items (Format)***	***Scoring (Range)***	***Item Example***
Attitude:	5 items/ 5 point Likert Scale (strongly disagree- strongly agree)	^a^Strongly Disagree = 1, Disagree = 2, No idea = 3, Agree = 4, Strongly Agree = 5 5–25	I think dentists care only about treatment and not prevention
Subjective norms:	4 items / 5 point Likert Scale (strongly disagree- strongly agree)	SD = 1, D = 2, NI = 3, A = 4, SA = 5 4–20	The people who are important to me (such as family) think I should brush my teeth every day
perceived behavioural control	4 items/ 5 point Likert Scale (strongly disagree- strongly agree)	SD = 1, D = 2, NI = 3, A = 4, SA = 5 4–20	I am confident that I can perform my own oral health self-care
Intention	4 items/ 5 point Likert Scale (strongly disagree- strongly agree)	SD = 1, D = 2, NI = 3, A = 4, SA = 5 4–20	I intend to brush my teeth regularly over next month

^a^ *SD* Strongly Disagree, *D* Disagree, *NI* No Idea, *A* Agree, *SA* Strongly Agree

### Measurements

#### Measures of behavioral intention

By the measures of behavioral intention, the probability that participants frequently performed specific oral health behaviors was evaluated, using a 5-point Likert scale (1) extremely unlikely and (5) extremely likely. The intention items included: I will brush my teeth more than twice per day, I will floss my teeth every day during the next month, I will use mouthwash daily, I will visit the dentist on a frequent schedule, and I will undergo dental scaling on a regular basis. The Cronbach’s alpha of the scale obtained 0.89. (Table 3).

**Table 3:** TPB Questionnaire

***Construct***	Items	***Scoring***
Attitude:	If my teeth decay, I will get severe toothache.	Strongly Disagree = 1, Disagree = 2, No idea = 3, Agree = 4, Strongly Agree = 5
	If my teeth decay, I will look ugly.	
	Brushing my teeth makes my teeth whiter.	
	At the late night and when I want to sleep, I am tired and do not brush/ floss my teeth.	
	I don’t have a toothbrush with me at work or university and I don’t brush.	
Subjective norms:	My family believe that I should brush/ floss my teeth.	Strongly Disagree = 1, Disagree = 2, No idea = 3, Agree = 4, Strongly Agree = 5
	My friends believe that I should brush/ floss my teeth.	
	Social pressure makes me to engage in oral health behavior.	
	When I am with my colleagues, I will brush/ floss even out of compulsion	
perceived behavioural control	It is difficult to me to engage in the oral health behavior such as brushing/flossing my teeth every day.	Strongly Disagree = 1, Disagree = 2, No idea = 3, Agree = 4, Strongly Agree = 5
	If I intended to, I would not have problems successfully engaging in the oral health behavior such as brushing/flossing my teeth every day.	
	I have complete control over engaging the oral health behavior such as brushing/flossing my teeth every day.	
	It is completely depending on me whether I engage in the oral health behavior such as brushing/flossing my teeth every day.	
Intention	I intend to brush/ floss my teeth twice a day.	Strongly Disagree = 1, Disagree = 2, No idea = 3, Agree = 4, Strongly Agree = 5
	I intend brush/ floss twice a day.	
	I will try to rinse my mouth at least with water after consuming sweets.	
	I decide to see a dentist every 6 months to have my teeth examined.	

#### Affective attitude toward the behavior

Two items evaluated the affective relationships with oral health behaviors. Participants were asked to indicate how they felt when thinking of orderly tooth brushing, flossing, dental visits, and scaling (unpleasant/pleasant) and replied using a 5-point Likert scale with 1 and 5 considered at each end. The Cronbach’s alpha of the scale obtained 0.88. The mean of the five items as the measure of affective attitude was applied. (Table 3).

#### Cognitive attitude toward the behavior

The attitudes toward oral health behaviors were evaluated with three items that measured the anticipated value of engaging in frequent oral health behaviors. Each question included a semantic differential (harmful/beneficial) anchoring each end of a 5-point response scale conforming the prompt, “For me [engaging the oral health behavior such as flossing my teeth daily on a regular basis is …” . The mean of the items as the total measure of cognitive attitudes was applied (α = 0.88). (Table 3).

#### Subjective norms

Four items for each behavior were applied, assessed by 5-point scales, to evaluate subjective norms, namely, “Most people who are important to me would like me to engage the oral health behavior such as flossing my teeth every day” and “I feel social pressure to apply preventive oral health behaviors such as flossing my teeth every day” (disagree completely/agree completely). The mean of the items as the measure of social norms was applied (α = 0.93). (Table 3).

#### Perceived behavioral control

Perceived behavioral control (PBC) for each behavior was evaluated using four indicators, all assessed by 5-point Likert scales. One item measured the perceived difficulty of engaging the behavior: “For me it is difficult to [apply preventive oral health behaviors such as flossing my teeth every day” (disagree completely/agree completely). One question measured the degree that the participants were confident (CON) that they would be able to successfully engage in the behavior: “If I wanted to, I would not have problems successfully [applying preventive oral health behaviors such as flossing my teeth every day” (disagree completely/agree completely). One item demonstrated the perceived control (PC): “I have full control over applying preventive oral health behaviors such as flossing my teeth everyday” (disagree completely/agree completely). Ultimately one item measured the locus of control (LOC): “It is thoroughly up to me whether I apply preventive oral health behaviors such as flossing my teeth every day” (disagree completely/agree completely). The mean of the items as the measure of PBC was used (α = 0.71). (Table 3).

### Dental examination (BOP and DMFT index)

Calculating and completing demographic information and determining the number of decayed, missing and filled teeth (DMFT) was completed by a dentist, in a room with sufficient lighting and using a torch to help illuminate the oral environment and to improve documentation. The periodontal examination calculated DMFT, BOP and the periodontal screening index (PSI). Caries prevalence were analyzed applying the DMFT indices. The BOP index was used to identify gingivitis and other inflammation. Each tooth was examined and the BOP index was computed by the number of bleeding spots divided by all measured spots. The PSI for both groups were specified in order to determine requirements for periodontal treatment [47]. For each participant DMFT index was recorded using the guidelines of the World Health Organization (WHO) [48]. An oral care clinical consultant conducted the dental examinations and included a full-mouth assessment of number of remaining teeth and assessment of periodontal status. The periodontal status was defined as Periodontal Screening Index (PSI) applying a WHO probe [49]. PSI was defined as follows: Score 0 showed healthy, score 1 bleeding, score 2 supra−/sub gingival dental calculus, and score 3 probing depths from 3.5 mm to max. 5.5 mm, and score 4 probing depth greater than 5.5 mm [50]. We also specified “No periodontitis” as a score under 3, and “periodontitis” as a score of 3 or more. In addition to the PSI score, the bleeding on probing (BOP) was determined as a solitary variable and the existence of plaque was recorded whenever soft or hard plaque was observed during the dental examination.

The DMFT index was applied, using flat dental mirrors and sharp sickle–shaped explorers.

### Study procedure

Ethics approval from both the hospital and the university committees were obtained and hospital wards provided assurance of participant confidentiality. Appropriate private space for the completion of interviews was also provided. Post intervention, training was then also provided for the control group.

#### Intervention

Pre-test questionnaires were administered to both groups at the start of the study. The pre-test results were used to develop an educational needs analysis including the training/education package and the number of sessions required. The education sessions for the intervention group were conducted on each ward for 2 months. The content of each training session was arranged based on the needs analysis, considering previous peer reviewed sources as well as oral health field specialists’ points of view. After developing the pre-test materials (pamphlets, PowerPoint slides, etc.) these were piloted with 10 participants. Pilot participants were chosen by convenience and their data were not included in the analysis performed for the study.

Three 90-min training/education sessions were designed in the form of lectures and with PowerPoint presentations. The intervention was applied for 1 month and follow up occurred 2 months after the intervention.

The educational method was didactic but included discussion. In the sessions individuals were encouraged to discuss their beliefs and attitudes towards oral health among staffs, their own brushing and flossing techniques as well as general oral and dental hygiene issues. Discussion included limitations to oral health behavior change despite difficult living conditions. Formal lectures and discussion were used together with dental replica models. The importance of oral hygiene among staff and the impact that environmental surroundings have on oral hygiene were discussed. All participants were provided with, an oral health education leaflet. The fourth session aimed at reviewing the content of previous sessions and re-emphasizing the impact of perceived behavior control on oral health behavior. After reviewing the content from the previous sessions, the facilitator again discussed the impact of the broader environment has on preventing good oral health while working in the hospital.

#### Data an2alysis

Data were analyzed with SPSS V.21, applying descriptive analytics including mean, standard deviation, frequency and percentages. The normality of distribution parameters was evaluated by Shapiro-Wilk test. The inferential statistics also was applied using the independent-samples T-test, Repeated-Measures one-way ANOVA, and matched T-test. The significance level was set at *p* < 0.05.

## Results

Participation in the study was very good with most people in both groups attending all stages of the research. Seven participants were excluded (four participants from the experimental group and three from the control group, three did not complete the questionnaire and four did not provide complete responses). In addition, having the researcher present while participants completed the questionnaires ensured there was minimal missing data. The total sample included 76 participants in the intervention arm of the study and 77 in the control group.

The average age of participants in the intervention group was 36.2 ± 8.3 years and for the control group it was 37 ± 8.42 years. T-test and Chi-squared tests considered the homogeneity of the research variables and demonstrated no statistically significant difference (*p*>.05) between the two groups in terms of age, sex, education, marital status, and income status (see Table 4). Findings revealed significant variations between the groups in the first post-test (immediately after the educational intervention) concerning the attitudes, subjective norms, perceived behavioral control (PBC), intentions, oral health behavior as well as DMFT and BOP (*p*<0.001). Moreover, 2 months after the intervention, except for the brushing construct (*p* = .18), differences between the two groups were statistically significant in all of the other constructs (*p*<0.001) (see Table 5).

**Table 4:** Comparison of demographic variables between the both groups

***variable***		***Control*** N (%)	***Experimental*** N (%)	***P-value***
Age	< 30 Years	12 (18)	14 (21)	0.84
	30–50 years	48 (71.6)	45 (67.1)	
	> 50 years	7 (10.4)	8 (11.9)	
Sex	Male	33 (49.3)	38 (57.5)	0.22
	Female	34 (50.7)	28 (42.5)	
Education	Bachelor of science	34 (50.7)	31 (47)	0.87
	Master of sciences	23 (34.3)	25 (38)	
	PhD	10 (14.9)	10 (15)	
Marital status	Single	48 (71.6)	42 (64)	0.76
	Married	19 (28.4)	24 (36)	
**income status**	< 250$	13 (19.4)	15 (22.7)	0.65
	250–400$	31 (46.3)	29 (44)	
	> 400$	23 (34.3)	22 (33.3)

**Table 5:** Comparison of the TPB construct scores between both groups before,immediately after and 2 months after the education intervention

Variable (range)	Group	Before intervention	Immediately after intervention	2-month after intervention	***P***.value
		Mean ± SD	Mean ± SD	Mean ± SD	
**Brushing**	***Experimental***	**1.73 ± 0.64**	**1.84 ± 0.85**	**1.82 ± 0.76**	**0.18**
	***Control***	**1.66 ± 0.78**	**1.66 ± 0.74**	**1.63 ± 0.85**	**0.14**
	*p-*value*	**0.17**	**< 0.001**	**< 0.001**	
	**Mean difference**	**0.18**	**0.14**	**0.19**	
**Visit of dentists**	***Experimental***	**1.49 ± 0.59**	**1.76 ± 0.48**	**1.7 ± 0.58**	**< 0.001**
	***Control***	**1.51 ± 0.59**	**1.51 ± 0.52**	**1.49 ± 0.54**	**0.06**
	*p-*value*	**0.7**	**< 0.001**	**< 0.001**	
	**Mean difference**	**−0.02**	**0.25**	**0.21**	
**Dental flossing**	***Experimental***	**1.38 ± 0.48**	**1.62 ± 0.48**	**1.72 ± 0.38**	**< 0.01**
	***Control***	**1.34 ± 0.43**	**1.53 ± 0.50**	**1.66 ± 0.52**	**< 0.01**
	*p-*value*	**0.08**	**< 0.01**	**< 0.001**	
	**Mean difference**	**0.04**	**0.09**	**0.06**	
Attitude 5–25	***Experimental***	**19.33 ± 1.52**	**21.25 ± 3.3**	**20.72 ± 3.3**	**0.04**
	***Control***	**19 ± 1**	**17 ± 2.58**	**16.75 ± 3.22**	**0.3**
	*p-*value*	**0.66**	**< 0.01**	**< 0.001**	
	**Mean difference**	**0.33**	**4.25**	**−0.03**	
Subjective norms 4–20	***Experimental***	**12.4 ± 2.07**	**15.8 ± 2.38**	**15.55 ± 1.86**	
	***Control***	**12.6 ± 1.14**	**12.52 ± 1.52**	**12.22 ± 1.48**	**0.16**
	*p-*value*	**0.74**	**< 0.01**	**< 0.01**	
	**Mean difference**	**−0.2**	**0.28**	**0.33**	
perceived behavioral control 4–20	***Experimental***	**9.8 ± 1.92**	**14.4 ± 2.3**	**14.2 ± 2.1**	**< 0.01**
	***Control***	**9.6 ± 1.14**	**9.6 ± 1.81**	**9.2 ± 1.78**	0.19
	*p-*value*	0.81	**< 0.03**	**< 0.02**	
	**Mean difference**	0.2	4.8	5	
intention 4–20	***Experimental***	**8.24 ± 3.5**	**14 ± 2.94**	**13.98 ± 1.94**	**<** 0.02
	***Control***	**9 ± 2.1**	**8.42 ± 2.87**	**8.12 ± 2.93**	0.36
	*p-*value*	0.62	**<** 0.02	**<** 0.02	
	**Mean difference**	−0.76	5.58	5.86	
DMFT	***Experimental***	**2.311 ± 0.29**	**2.32 ± 0.28**	0.01 **± 0.01**	**0.6**
	***Control***	**2.32 ± 0.28**	**2.33 ± 0.27**	0.01 **± 0.01**	0.4
	***p-*value**	0.06	0.7	**0.8**	
	**Mean difference**	0.01	0.01	0.00	
BOP PSI (min 0–max 5.5 mm)	***Experimental***	**2.05 ± 0.07**	**1.89 ± 0.31**	**1.90 ± 0.32**	**<** 0.01
	**control**	**2.04 ± 0.04**	**2.94 ± 0. 32**	**2.91 ± 0. 29**	0.25
	*p-*value*	0.74	**<** 0.01	**<** 0.01	
	**Mean difference**	0.01	−1.05	−1.01	

Mean differences in scores were larger immediately after the intervention than at 2 months post-intervention. The difference in DMFT of participants was greatest immediately after the intervention (see Table 5). For the intervention group, the mean difference before, immediately after and 2 months post intervention was 0.01, 0.01and 0.00respectively. Concerning the BOP index and PSI, our results illustrate a remarkably lower risk for gingivitis and periodontitis among the experimental group. When comparing the mean difference of the intervention and control groups before, immediately after and 2 months’ post intervention was 0.01, − 1.05 and − 1.01 respectively. Further, in comparison with the control group, the intervention group showed enhanced oral health behaviors on all three measures (Table 5). The mean differences respectively for brushing in the intervention and control groups before, immediately after and 2 months after the intervention were (0.18, 0.14, 0.19); for visiting the dentist (− 0.02, 0.25, 0.21); and for dental flossing (0.04, 0.09, 0.06).

## Discussion

Using a framework informed by the TPB, this study aimed to investigate the effect of an educational intervention on the oral health behaviors, DMFT and BOP index among hospital staff in Tehran. The study measured the impact of the intervention on dental flossing, brushing and frequency of dental visits. Mixed results exist in previous research on the best behavioral interventions to improve oral health behavior in individuals with poor oral health [51, 52]. Positive effects on tooth-brushing and flossing have been shown with a randomized control trial (RCT) of middle-aged and older individuals with periodontitis [53–55]. Encouragingly our educational intervention with hospital staff enhanced their oral health behavior on all examined variables (tooth-brushing, flossing, and visiting the dentist). All participants had severe tooth decay, and behavioral change was required to slow disease progression and to improve overall oral health. The data show a significant difference for the intervention arm when comparing the attitude scores before, immediately after and in the 2 months after the education sessions.

In line with previous research our findings show a positive relationship between parental oral health knowledge, attitudes, and perceived behavioral control and their intention [19]. The finding that the benefits of the intervention were more profound in individuals with a practical mindset, i.e., those who were more likely to report an intention to brush their teeth regularly in the next month confirms the importance of goal setting and goal achievement. If participants report an intention to change their behaviors, then developing programs that facilitate when, where and how to apply preventive oral health behavior change can occur may have more success. If participants report no intentions to change then forcing them to construct plans is unlikely to facilitate behavior change and even may cause resistance. This must be kept in mind when designing future oral health behavioral interventions because such interventions are likely only to benefit individuals who are already considering a change in behavior. Previous research documenting the differential impacts of self-monitoring for dental flossing further emphasizes the idea of different mindsets in individuals [56]. For participants yet to make a decision to change other approaches might be more influential, e.g. giving information about the symptoms, causes, consequences, temporal course and prevention activities for periodontal disease [57]. Furthermore, matching appropriate interventions to the specific needs of individuals has previously been shown to have a positive impact on oral hygiene behavior [58].

In our multi-disciplinary experimental research, dental personnel identified eligible attendees, and the intervention was performed by a psychologist working at the same general dental clinic. While the inclusion of psychologists in primary care environments has become more common [59] this has not been adopted widely in dentistry, except with the treatment of patients with dental phobia in specialist clinics [59, 60]. Due to the positive health benefits that can be achieved by adjusting oral health behaviors, eliminating the structural barriers of the health system (such as lack of insurance support, dentist inaccessibility, and high cost of service) it is recommended that policies be put in place that facilitate increasing insurance coverage and the general accessibility to oral health care. The development of education programs, especially among hospital staff, that emphasize oral hygiene are worthy of further investigation.

The strengths of this study include the application of the theory-based model in the study and the provision of an intervention for hospital staff that considered multiple oral health-related behaviors. Limitations include participant self-reporting and the potential for social desirability bias which may have seen an overestimation of positive health behaviors reported in some parts of the survey. To reduce and minimize this bias, the anonymous questionnaires were used. With few previous studies completed in this area comparison was difficult. The lack of comprehensive focus on broader contextual factors is also a limitation of using the TPB as a framework. The limited follow-up post intervention means the positive results shown with regard to oral health behavior may not have been sustained beyond the 2 month follow-up period. Also, DMFT and PSI are not the ideal dental measures in interventional studies due to low capacity to discriminate the conditions between groups and over time.

## Conclusion

Our findings clarify the positive effect of education on enhancing the attitude, subjective norms, perceived behavioral control, intention, and the behavior of hospital staff. The results of the present study highlight that developing and applying an educational intervention informed by the TPB framework can lead to significant changes in the knowledge, attitudes, and performance of staff regarding preventive measures for tooth decay. Policymakers and oral health educators must make the most of opportunities to develop behavioral model interventions to influence oral/dental health. Providing support to hospital staff to be conscious of the importance of performance-based healthy behaviors is recommended. This can enhance the effectiveness of the oral health educational intervention and potentially improve preventive behaviors. Our findings support other studies showing that the application of well-designed models such as the TPB and appropriate educational programs in the hospital ward setting may help to improve healthier dental/oral behaviors. To have more impact and to increase sustainability oral health education programs must be combined with regular community based oral health initiatives.

## Data Availability

The dataset used during the study are available from the corresponding author upon request.

## Abbreviations

TPB: Theory of planned behavior
CVR: Content validity ratio
CVI: Content validity index used Content Validity Index
ICC: Intraclass correlation coefficient
CON: Confident
PC: Perceived control
LOC: Locus of control
WHO: World Health Organization
DMFT: Decayed, Missing, and Filled Teeth
BOP: Bleeding on probing
PSI: Periodontal screening index
PBC: Perceived behavioral control
RCT: Randomized control trial
